# Structural Lung Disease in Children and Adolescents With Severe Neurological Disorders

**DOI:** 10.1002/ppul.71698

**Published:** 2026-06-10

**Authors:** Daniel A. F. Bernard, Cathrin Dahl, Luca Salhöfer, Anna Pauly, Mathis Steindor, Margarete Olivier, Ulrike Schara‐Schmidt, Matthias Welsner, Bernd Schweiger, Sebastian Zensen, Marcel A. Drews, Johannes Haubold, Michael Forsting, Lale Umutlu, Marcel Opitz, Florian Stehling

**Affiliations:** ^1^ Pediatric Pulmonology and Sleep Medicine, University Hospital Essen University of Duisburg‐Essen Essen North Rhine‐Westphalia Germany; ^2^ Institute of Diagnostic and Interventional Radiology and Neuroradiology University Hospital Essen Essen North Rhine‐Westphalia Germany; ^3^ Department of General Pediatrics, Neonatology, and Pediatric Cardiology, University Children's Hospital, Medical Faculty Heinrich Heine University Duesseldorf North Rhine‐Westphalia Germany; ^4^ Department of Pediatric Neurology, Center for Neuromuscular Disorders in Children and Adolescents, University Hospital Essen University of Duisburg‐Essen Essen North Rhine‐Westphalia Germany; ^5^ Department of Pulmonary Medicine University Hospital Essen‐Ruhrlandklinik Essen North Rhine‐Westphalia Germany

**Keywords:** bronchiectasis, Chest computed tomography (CT), dysphagia, neurologic disorders, neuromuscular diseases

## Abstract

**Background and Objective:**

Children with severe neurological disorders are at risk of secondary respiratory morbidity due to impaired airway clearance and dysphagia, but systematic data on structural lung changes remain scarce.

**Methods:**

We retrospectively analyzed all clinically indicated chest CT examinations at a tertiary care center (2015–2025) in 34 children with severe neurological disorders (median age 10 years), excluding those with primary lung disease. Exploratory hierarchical clustering identified clinical phenotypes based on disease etiology, respiratory support, dysphagia, and mobility.

**Results:**

Structural abnormalities were common. Lobar consolidation (76%) and bronchial wall thickening (62%) were the most frequent CT findings. Cluster analysis identified three phenotypes: a stable neuromuscular phenotype without bronchiectasis or *Pseudomonas aeruginosa* colonization, an advanced neuromuscular‐dysphagic phenotype with the highest bronchiectasis prevalence (64%, *p* = 0.005), and a neurologic‐dysphagic phenotype characterized by frequent consolidations (86%) and ground‐glass opacities (36%). Although most scans were elective (76%), CT prompted management changes in 91% of patients, mainly intensified airway clearance, antibiotic treatment, and escalated respiratory support. Among CT abnormalities associated with subsequent management change, 60% were not clearly identifiable on retrospectively reviewed preceding chest radiographs.

**Conclusion:**

In this selective cohort of children with severe neurological disorders undergoing clinically indicated chest CT, structural lung abnormalities were common despite the absence of a primary pulmonary diagnosis. Exploratory clinical phenotyping suggested distinct risk patterns linked to dysphagia, impaired airway clearance, and microbial colonization, and may help identify patients in whom CT provides clinically relevant additional information. Prospective studies are warranted before broader CT‐based strategies can be recommended.

## 1 Introduction

The cohort of children living with long‐term mechanical ventilation due to chronic respiratory insufficiency has increased continuously [1, 2, 3, 4]. Most of these children suffer from severe neurological impairment, representing a highly vulnerable cohort where lung morbidity is secondary to functional deficits rather than a primary pulmonary condition [5]. The main indication for respiratory surveillance is progressive respiratory muscle weakness resulting in hypoventilation and cough insufficiency [6, 7]. However, the progressive underlying neurological disease regularly results in progression of the respiratory muscle weakness with impaired clearance of airway secretions [8]. Insufficient airway clearance is often aggravated by dysphagia with insufficient swallowing resulting in (micro‐)aspiration [9, 10]. Chronic increase of airway secretion may result in chronic bronchitis with neutrophilic inflammation and colonization/infection with potential pathogenic bacteria like *Pseudomonas aeruginosa* [11]. In children with tracheostomy, this pathophysiological sequence has been demonstrated in multiple studies [12, 13]. In addition, progressive thoracic restriction and cough insufficiency themselves seem to be risk factors for bacterial airway colonization [14]. In otherwise healthy children, the presence of chronic wet cough and retention of airway secretions is labeled protracted bacterial bronchitis – a condition that is believed to be the precursor of bronchiectasis in children [15, 16]. Conditions of severe neurological impairment, like neuromuscular disease (NMD) or cerebral palsy, are frequently associated with airway colonization with pathogenic bacteria and impairment of swallowing [14]. Dysphagia itself was recently identified as a risk factor for airway colonization with potentially pathogenic bacteria [17, 18]. Especially the presence of gram‐negative bacteria is regarded a risk factor for major respiratory complications [18]. Systematic data on chronic (secondary) structural lung disease in children with neurological disorders are sparse. Previous multiple‐breath washout studies in Duchenne muscular dystrophy and spinal muscular atrophy have shown that greater ventilation inhomogeneity is associated with more advanced restrictive ventilatory impairment [19, 20]. The literature provides a single report on chest CT imaging in eight DMD patients indicating that microatelectasis may be associated with thoracic restriction [21]. With this retrospective analysis, we aim to describe which structural lung disease conditions are present in children with severe neurological disorders.

## 2 Methods

### 2.1 Patients

This analysis included all chest computed tomography (CT) examinations performed between 2015 and 2025 during clinical assessments at the University Hospital Essen in patients with severe neurological disorders characterized by sustained motor and/or bulbar dysfunction, requiring coordinated follow‐up in Pediatric Neurology and Pediatric Pulmonology. Patients with known primary respiratory diseases were excluded. The study was approved by the local Ethics Committee (25‐12617‐BO) and reported in accordance with STROBE guidelines for observational studies.

### 2.2 Chest Computed Tomography

**Figure 1:**
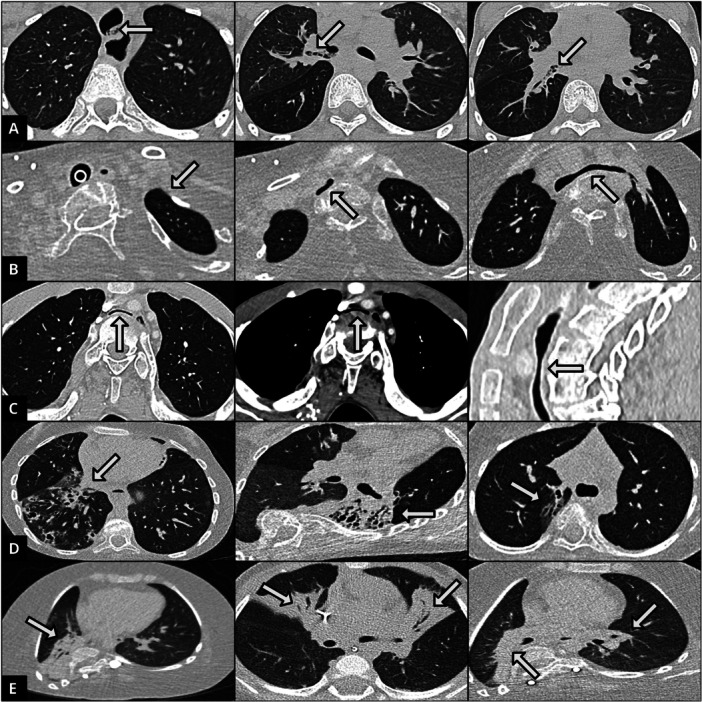
Representative chest computed tomography (CT) findings in children with severe neurological disorders. (A–C) CT scans from a single patient demonstrating retained secretions within the central tracheobronchial tree (A) and subsequent tracheal stenosis secondary to severe thoracic deformity (B, C). (D) Bronchiectasis in a second patient. (E) Lung consolidations in a third patient. Pathological findings are indicated by arrows.

Chest CT was performed with or without intravenous contrast, depending on clinical indication, using either a NAEOTOM Alpha or a dual‐source 128‐slice SOMATOM Definition Flash (Siemens Healthineers). Automatic tube current (CARE Dose 4D) and voltage modulation (CARE kV, down to 70 kV) were applied to minimize radiation exposure. Consolidation was defined collectively as reduced lung ventilation patterns, including dystelectasis and segmental or lobar atelectasis. Pneumonia could not be excluded radiologically and was therefore subsumed under this definition. All CT scans and preceding chest radiographs (CXR) were re‐reviewed retrospectively by board‐certified radiologists with experience in thoracic imaging using a predefined structured item list. Representative examples of CT abnormalities assessed in this study are provided in Figure 1. Because of the retrospective design, reviewers were not systematically blinded to the clinical context, and formal inter‐rater reliability analysis was not performed.

### 2.3 Clinical and Microbiological Definitions

Data were extracted via chart review. In this context, chronic respiratory insufficiency refers to sustained ventilatory failure and/or ineffective airway clearance requiring long‐term respiratory support and structured multidisciplinary follow‐up. Clinically relevant dysphagia was defined as documented swallowing dysfunction leading to feeding modification, tube feeding, or specific therapeutic intervention. Classification was based on standardized clinical assessments by specialized speech‐language pathologists. Instrumental swallowing studies were not consistently available, and objective aspiration was therefore not systematically ascertainable. Microbiological airway colonization was defined as the repeated culture‐positive detection of typical pathogens in routine airway secretions. Samples consisted of throat swabs in non‐tracheostomized patients and tracheal aspirates in tracheostomized patients. Microbiological analyses were performed in the institutional microbiology laboratory according to routine accredited procedures. Because of the retrospective design, microbiological sampling was not standardized in relation to CT timing. Given the cohort's vulnerability, organisms such as *Pseudomonas aeruginosa*, *Staphylococcus aureus*, and *Stenotrophomonas maltophilia* were consistently categorized as potentially pathogenic.

### 2.4 Statistics

**Figure 2:**
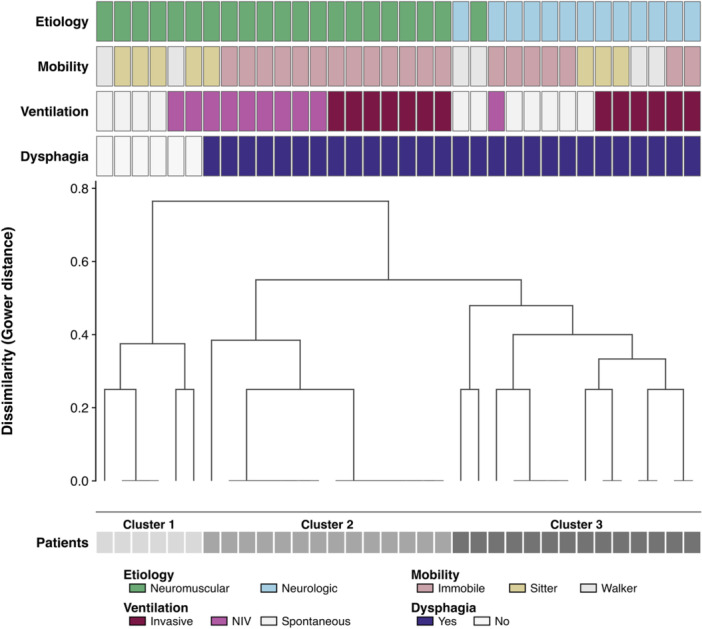
Clinical phenotyping via unsupervised hierarchical clustering. Agglomerative hierarchical clustering was performed using the average‐linkage method and a Gower distance matrix. Each column represents one patient. Colored annotation bars show the four cluster‐defining variables: etiology, respiratory support mode, dysphagia, and mobility, with their respective subcategories indicated in the figure key. The dendrogram illustrates an exploratory three‐cluster solution corresponding to three clinically interpretable phenotypes: Cluster 1, a stable neuromuscular phenotype with preserved bulbar function; Cluster 2, an advanced neuromuscular‐dysphagic phenotype; and Cluster 3, a predominantly central neurologic‐dysphagic phenotype. [Color figure can be viewed at wileyonlinelibrary.com]

Statistical analyses were performed using R (version 4.5.0; R Foundation for Statistical Computing, Vienna, Austria). Continuous variables are presented as medians with ranges and categorical variables as absolute and relative frequencies. To explore whether clinically interpretable phenotypes could be identified within this heterogeneous cohort, an unsupervised machine learning approach was employed. Agglomerative hierarchical clustering was performed using the average‐linkage method based on a Gower distance matrix. The model was built upon four core clinical, cluster‐defining variables: disease etiology, respiratory support mode, presence of clinically relevant dysphagia, and mobility status. The resulting hierarchical structure was visualized using a dendrogram (Figure 2). To assess the quality and robustness of the clustering solution, we calculated the Average Silhouette Width (ASW) and performed a stability analysis using bootstrapping (*B* = 100). Cluster stability was quantified using the mean Jaccard similarity index, with values > 0.75 indicating highly stable patterns. Phenotypes were compared using Kruskal–Wallis tests for continuous variables and Fisher's exact tests for categorical data. Reported overall *p*‐values represent omnibus comparisons across all three clusters. Statistically significant overall results (*p* < 0.05) were followed by Holm–Bonferroni‐corrected pairwise post‐hoc comparisons. No inferential *p*‐values were reported for variables used to derive the clustering solution, as these variables contributed to cluster assignment. Given the small sample size, all subgroup comparisons were exploratory, and no multivariable modelling was performed.

## 3 Results

### 3.1 Study Population

Between 2015 and 2025, 34 patients with severe neurological disorders, including neuromuscular (*n* = 21) or other neurological conditions (*n* = 13) underwent chest CT imaging. The underlying diseases were heterogeneous and are summarized in E‐Table 1. Except for three patients who initially presented during an acute illness, all patients had been followed for many years. A detailed overview of patient characteristics is provided in Table 1. Median age at CT was 10 years (range: 3 months–17 years). At the time of assessment, 13 patients were tracheostomized and required at least intermittent invasive ventilation, and 10 received non‐invasive ventilation (NIV). A mechanical insufflation‐exsufflation (MI‐E) device was used by 23 patients. Airway colonization was common, particularly in dysphagic and tracheostomized patients, with *Pseudomonas aeruginosa* being the predominant organism. To structure this highly heterogeneous cohort and identify biologically relevant subgroups, an unsupervised hierarchical cluster analysis was performed based on four core clinical variables: disease etiology (neuromuscular vs. neurologic), respiratory support mode (invasive ventilation, non‐invasive ventilation, or spontaneous breathing), clinically relevant dysphagia (yes vs. no), and mobility status (immobile, sitter, or walker). This yielded three exploratory clinical phenotypes (Table 1) that were clinically interpretable and showed acceptable internal separation and high bootstrap stability. The three‐cluster solution demonstrated a solid internal structure with an overall Average Silhouette Width of 0.52 (Cluster 1: 0.61; Cluster 2: 0.68; Cluster 3: 0.33) (E‐Figure 1). Furthermore, the stability analysis confirmed that these phenotypes are highly robust, yielding mean Jaccard similarity indices of 0.90 for Cluster 1, 0.87 for Cluster 2, and 0.82 for Cluster 3, significantly exceeding the standard stability threshold of 0.75. Cluster 1 represented a comparatively stable neuromuscular phenotype without dysphagia or chronic *Pseudomonas aeruginosa* colonization. Cluster 2 captured an advanced neuromuscular‐dysphagic phenotype with profound immobility, high respiratory support requirements, and the highest burden of bronchiectasis. Cluster 3 comprised predominantly central neurologic disorders with dysphagia and frequent consolidative CT changes rather than bronchiectasis. Anthropometric profiling demonstrated pervasive nutritional wasting across the entire study population, with an overall median weight z‐score of −1.14. When stratified by the algorithmically derived phenotypic clusters, weight z‐scores remained uniformly depressed without statistically significant variance between the cohorts.

**Table 1:** Patient demographics, clinical characteristics, and microbiology stratified by phenotypic clusters.

Characteristic	Overall (*N* = 34)^a^	Cluster 1 (*n* = 6)^a^	Cluster 2 (*n* = 14)^a^	Cluster 3 (*n* = 14)^a^	*p* b
**Demographics & Anthropometrics**					
** Sex**					0.51
* Female*	11 (32%)	2 (33%)	3 (21%)	6 (43%)	
* Male*	23 (68%)	4 (67%)	11 (79%)	8 (57%)	
** Age (years)**	10.0 (0.0–17.0)	12.0 (7.0– 16.0)	9.5 (3.0–16.0)	11.0 (0.0–17.0)	0.57
** BMI**	15.4 (8.7–32.7)	21.7 (11.0– 25.8)	14.6 (11.8– 25.5)	15.9 (8.7–32.7)	0.32
** Weight (z‐score)**	−1.14 (−15.46– 3.72)	−1.55 (−2.80– 1.53)	−1.12 (−4.86– 2.23)	−0.97 (−15.46– 3.72)	> 0.99
**Cluster‐defining variables (descriptive)**					
** Etiology**					
* Neurologic*	13 (38%)	0 (0%)	0 (0%)	13 (93%)	
* Neuromuscular*	21 (62%)	6 (100%)	14 (100%)	1 (7.1%)	
** Resp. support mode**					
* Invasive ventilation*	13 (38%)	0 (0%)	7 (50%)	6 (43%)	
* Non‐invasive ventilation*	10 (29%)	2 (33%)	7 (50%)	1 (7.1%)	
* Spontaneous breathing*	11 (32%)	4 (67%)	0 (0%)	7 (50%)	
** Dysphagia**	28 (82%)	0 (0%)	14 (100%)	14 (100%)	
** Mobility**					
* Immobile*	20 (59%)	0 (0%)	13 (93%)	7 (50%)	
* Sitter*	8 (24%)	4 (67%)	1 (7.1%)	3 (21%)	
* Walker*	6 (18%)	2 (33%)	0 (0%)	4 (29%)	
**Additional clinical features**					
** Tracheostomy**	13 (38%)	0 (0%)	7 (50%)	6 (43%)	0.10
** MI‐E**	23 (68%)	3 (50%)	14 (100%)	6 (43%)	**0.002 (1 vs. 2: *p*** = **0.035) (2 vs 3: *p*** = **0.006)**
** PEG**	22 (65%)	1 (17%)	11 (79%)	10 (71%)	**0.029**
** Scoliosis**	19 (56%)	4 (67%)	7 (50%)	8 (57%)	0.90
**Microbiology**					
* Pseudomonas aeruginosa*	23 (68%)	0 (0%)	12 (86%)	11 (79%)	**< 0.001 (1 vs. 2: *p*** = **0.002) (1 vs. 3: *p*** = **0.004)**
* Stenotrophomonas maltophilia*	7 (22%)	0 (0%)	4 (29%)	3 (23%)	0.63

^a^ n (%); Median (Min ‐ Max).

^b^ Omnibus *p*‐values compare the three clusters simultaneously and were calculated using Kruskal–Wallis tests for continuous variables and Fisher's exact tests for categorical variables. Statistically significant pairwise post‐hoc comparisons (Holm‐Bonferroni corrected) are shown below the overall *p*‐value.

*Note:* The cohort (*N* = 34) was partitioned via hierarchical clustering based on etiology, respiratory support, dysphagia, and mobility.

Abbreviations: MI‐E = Mechanical insufflator‐exsufflator; PEG = Percutaneous endoscopic gastrostomy.

### 3.2 Indications for Imaging

Most CT examinations (76%) were performed electively rather than during acute deterioration. The main indications were chronic pulmonary symptoms (53%), acute respiratory deterioration (24%), and abnormal chest radiograph (CXR) (24%) (Table 3). CXR preceded CT in all patients. In the subgroup with CXR performed within 30 days before CT, retrospective re‐review suggested that the CT abnormality associated with subsequent management change was not clearly identifiable on the preceding radiograph in 60% of evaluable cases (E‐Figure 2).

### 3.3 Specific CT Findings

Structural abnormalities were common (Table 2). Lung consolidations (76%) were the most frequent finding, peaking in Cluster 3 (86%). Bronchial wall thickening was detected in 21 patients (62%). Notably, the presence of bronchiectasis (32%) differed significantly across the clinical phenotypes (*p* = 0.005). Bronchiectasis was most prevalent in Cluster 2 (64%), completely absent in Cluster 1, and significantly less frequent in Cluster 3 (14%). In line with this algorithmically derived pattern, bronchiectasis was exclusively found in patients with clinically relevant dysphagia, and all affected patients had a documented colonization with *Pseudomonas aeruginosa* during their preceding clinical course. Retention of airway secretions in the central airways was present in four patients (Figure 1A). A fluid‐filled esophagus was detected in four dysphagic patients, all of whom exhibited concomitant consolidations. Ground‐glass opacifications were most prevalent in Cluster 3 (36%). Furthermore, skeletal deformities of the thorax were a prevalent finding in this cohort of patients with severe neurological disorders (59%). Within this subgroup, scoliosis was most common (90%) and was frequently associated with bronchial obstruction, lung consolidation (85%) and diaphragmatic elevation (20%). In three cases tracheal or central bronchial stenosis occurred in connection with the thoracic deformity (Figure 1C).

**Table 2:** Specific structural lung and skeletal findings on chest computed tomography stratified by phenotypic clusters.

Characteristic	Overall (*N* = 34)^a^	Cluster 1(*n* = 6)^a^	Cluster 2 (n = 14)^a^	Cluster 3 (n = 14)^a^	*p* b
Bronchial wall thickening	21 (62%)	2 (33%)	11 (79%)	8 (57%)	0.17
Bronchiectasis	11 (32%)	0 (0%)	9 (64%)	2 (14%)	**0.005 (1 vs. 2: *p*** = **0.042) (2 vs. 3: *p*** = **0.042)**
Central airway stenosis	3 (8.8%)	1 (17%)	1 (7.1%)	1 (7.1%)	0.57
Central airway mucus	4 (12%)	0 (0%)	3 (21%)	1 (7.1%)	0.49
Mucus impaction	8 (24%)	1 (17%)	4 (29%)	3 (21%)	> 0.99
Lung consolidations	26 (76%)	3 (50%)	11 (79%)	12 (86%)	0.24
Ground‐glass opacification	6 (18%)	0 (0%)	1 (7.1%)	5 (36%)	0.14
Mosaic pattern	8 (24%)	2 (33%)	3 (21%)	3 (21%)	0.76
Skeletal deformities	20 (59%)	3 (50%)	9 (64%)	8 (57%)	0.90
Diaphragmatic elevation	5 (15%)	0 (0%)	2 (14%)	3 (21%)	0.82
Fluid‐filled esophagus	4 (12%)	0 (0%)	3 (21%)	1 (7.1%)	0.49

^a^ n (%).

^b^ Omnibus *p*‐values compare the three clusters simultaneously and were calculated using Fisher's exact tests for categorical variables. Statistically significant pairwise post‐hoc comparisons (Holm‐Bonferroni corrected) are shown below the overall *p*‐value.

**Table 3:** Clinical indications and setting for chest computed tomography examinations.

Characteristic	Overall (*N* = 34)^a^	Cluster 1(*n* = 6)^a^	Cluster 2 (*n* = 14)^a^	Cluster 3 (*n* = 14)^a^	*p* b
**CT setting**					0.32
*acute*	8 (24%)	0 (0%)	3 (21%)	5 (36%)	
*elective*	26 (76%)	6 (100%)	11 (79%)	9 (64%)	
**CT indication**					0.61
*abnormal findings in conventional chest radiography*	8 (24%)	2 (33%)	3 (21%)	3 (21%)	
*acute respiratory deterioration*	8 (24%)	0 (0%)	3 (21%)	5 (36%)	
*chronic pulmonary symptoms*	18 (53%)	4 (67%)	8 (57%)	6 (43%)	
**CT indication (detailed)**					0.42
*detection of potentially pathological microorganisms*	2 (5.9%)	0 (0%)	1 (7.1%)	1 (7.1%)	
*follow‐up of pneumothorax*	1 (2.9%)	1 (17%)	0 (0%)	0 (0%)	
*hypoxemia*	4 (12%)	0 (0%)	1 (7.1%)	3 (21%)	
*intensification of ventilation*	4 (12%)	1 (17%)	3 (21%)	0 (0%)	
*persistent wet cough*	4 (12%)	2 (33%)	1 (7.1%)	1 (7.1%)	
*pneumonia clinically suspected*	5 (15%)	0 (0%)	2 (14%)	3 (21%)	
*secretory problems*	2 (5.9%)	0 (0%)	2 (14%)	0 (0%)	
*status assessment*	1 (2.9%)	0 (0%)	0 (0%)	1 (7.1%)	
*suspected lung consolidation*	11 (32%)	2 (33%)	4 (29%)	5 (36%)	

^a^ n (%).

^b^ Omnibus p‐values compare the three clusters simultaneously and were calculated using Fisher's exact tests for categorical variables.

### 3.4 Diagnostic and Therapeutic Consequences

CT findings prompted a direct modification of clinical management in 91% of the overall cohort (Table 4). However, the impact varied by phenotype. While all of the patients in Cluster 3 received a direct change in therapy, Cluster 1 had the highest proportion of scans resulting in no immediate clinical consequence (33%). Bronchoscopy was performed in five patients. Two bronchoscopic procedures successfully resolved downstream atelectasis and associated CT‐morphologic consolidations resulting from secretion‐obstructed lung segments, as subsequently verified by ultrasound. Anti‐infective treatment was the most frequent therapeutic consequence. Short‐term antibiotic therapy was initiated or intensified in 11 patients (32%), predominantly in Cluster 3 (43%). Long‐term suppressive antibiotic therapy was established in nine patients (26%), most frequently in Cluster 2 (50%). In six previously orally fed patients, advanced CT abnormalities prompted an interdisciplinary reassessment of swallowing safety and aspiration risk in the context of the overall clinical course. Following this reassessment, PEG was recommended in three patients. Adjustments to airway clearance regimens, including the initiation or intensification of mechanical insufflation‐exsufflation (MI‐E), were recommended in 10 patients (29%). Long‐term ventilation was initiated or intensified in six cases (18%). In three patients presenting with severe scoliosis associated with critical airway compression or presumed ventilation deficits, the potential benefit of corrective spinal surgery was discussed. For one patient with cerebral palsy and epileptic encephalopathy, findings led to the implementation of palliative care.

**Table 4:** Diagnostic and therapeutic management changes prompted by chest computed tomography findings.

Characteristic	Overall (N = 34)^a^	Cluster 1(n = 6)^a^	Cluster 2 (n = 14)^a^	Cluster 3 (n = 14)^a^	*p*‐value^b^
Consequence derived from findings	31 (91%)	4 (67%)	13 (93%)	14 (100%)	0.074
MI‐E: Initiation	6 (18%)	3 (50%)	0 (0%)	3 (21%)	**0.020**
MI‐E: Intensification	4 (12%)	0 (0%)	2 (14%)	2 (14%)	> 0.99
Short‐term ABx: Initiation	8 (24%)	1 (17%)	3 (21%)	4 (29%)	> 0.99
Short‐term ABx: Intensification	3 (8.8%)	0 (0%)	1 (7.1%)	2 (14%)	> 0.99
Long‐term ABx: Initiation	9 (26%)	0 (0%)	7 (50%)	2 (14%)	**0.041**
Ventilation: Initiation	1 (2.9%)	1 (17%)	0 (0%)	0 (0%)	0.18
Ventilation: Intensification	5 (15%)	0 (0%)	4 (29%)	1 (7.1%)	0.23
Physiotherapy	5 (15%)	0 (0%)	3 (21%)	2 (14%)	0.82
Bronchoscopy: Diagnostic only	3 (8.8%)	1 (17%)	1 (7.1%)	1 (7.1%)	0.57
Bronchoscopy: Diagnostic and therapeutic	2 (5.9%)	0 (0%)	1 (7.1%)	1 (7.1%)	> 0.99
Recommendation for PEG	3 (8.8%)	0 (0%)	1 (7.1%)	2 (14%)	> 0.99
Recommendation for spinal surgery evaluation	3 (8.8%)	0 (0%)	0 (0%)	3 (21%)	0.20
Hypertonic saline (initiation)	1 (2.9%)	0 (0%)	0 (0%)	1 (7.1%)	> 0.99
Anticholinergics for drooling	1 (2.9%)	0 (0%)	0 (0%)	1 (7.1%)	> 0.99
Airway management equipment	1 (2.9%)	0 (0%)	0 (0%)	1 (7.1%)	> 0.99
Palliation	1 (2.9%)	0 (0%)	0 (0%)	1 (7.1%)	> 0.99
No immediate consequence	3 (8.8%)	2 (33%)	1 (7.1%)	0 (0%)	0.074

^a^ n (%).

^b^ Omnibus p‐values compare the three clusters simultaneously and were calculated using Fisher's exact tests for categorical variables. There were no statistically significant pairwise post‐hoc comparisons (Holm‐Bonferroni corrected). Abbreviations: ABx = Antibiotics; MI‐E = Mechanical insufflator‐exsufflator; PEG = Percutaneous endoscopic gastrostomy.

## 4 Discussion

This study describes CT‐detected structural abnormalities of the respiratory system and their clinical consequences in a pre‐selected, symptom‐driven cohort of children with severe neurological disorders without primary lung disease. In this selective high‐risk cohort, clinically relevant abnormalities of the lung parenchyma and thoracic cage were common and frequently associated with subsequent management changes. Exploratory cluster analysis suggested a coherent pathophysiological pattern in which dysphagia, impaired airway clearance, microbial colonization, and thoracic restriction interact to promote chronic secretion retention and secondary structural lung damage. Notably, structural abnormalities were not confined to acute deterioration, as most CT examinations were performed electively. Patients in Cluster 1 (a stable neuromuscular phenotype with preserved bulbar and motor function) exhibited neither bronchiectasis nor chronic *Pseudomonas aeruginosa* colonization. This pattern suggests that peripheral muscle weakness alone may be insufficient to drive chronic structural lung damage as long as swallowing function and basic airway clearance remain relatively preserved. A different pattern emerges when profound muscle weakness coincides with dysphagia (Cluster 2). This group exhibited the highest prevalence of bronchiectasis and chronic gram‐negative colonization. Severe bulbar dysfunction and profound peripheral muscle weakness likely represent parallel, mutually aggravating manifestations of progressive neuromuscular decline. Within this fragile cohort, patients may longitudinally transition along the clinical spectrum as their disease advances. Importantly, our data demonstrate that this structural deterioration may occur despite the implementation of non‐invasive airway clearance efforts, as all patients in this cluster were already prescribed MI‐E. This underscores that once progressive dysphagia and chronic micro‐aspiration manifest, mechanical cough assistance alone may not be sufficient to fully halt the progression toward bronchiectasis. A similar sequence has been described in other conditions associated with chronic wet cough, such as protracted bacterial bronchitis, cystic fibrosis, and primary ciliary dyskinesia, and is considered a precursor of bronchiectasis in children [22]. Chronic wet cough is associated with persistent neutrophilic inflammation and chronic bacterial colonization/infection by moisture‐dependent Gram negative bacteria like *Pseudomonas aeruginosa*. This vicious circle is aggravated by immobility and insufficient cough. Patients with primary central nervous system impairment and severe dysphagia (Cluster 3) shared a high *Pseudomonas aeruginosa* burden, but their structural disease was predominantly characterized by lobar consolidations and ground‐glass opacities rather than bronchiectasis. This reflects the dominance of bulbar dysfunction and acute mucus plugging over peripheral muscle weakness. In cough‐insufficient children, the inability to mobilize such thick mucus plugs can rapidly cause downstream airway obstruction and persistent infectious foci [6]. This leads to a pattern of “silent“ consolidations or localized ground‐glass changes on CT, which may remain clinically occult until a significant proportion of the lung is involved. Interestingly, despite the very high prevalence of lobar consolidations in this neurologic‐dysphagic phenotype, less than half of these patients were managed with an MI‐E device at the time of imaging. This significant discrepancy compared to the neuromuscular cohort highlights a potential therapeutic gap. It suggests that proactive mechanical airway clearance might be underutilized in children with primary central nervous system disorders. In this context, it is important to acknowledge that the implementation of proactive mechanical airway clearance in this population is often constrained by sparse evidence regarding clinical feasibility, tolerance, and efficacy [23]. The lower silhouette width of the neurologic‐dysphagic phenotype (0.33) reflects the inherent clinical heterogeneity of central nervous system disorders. However, the high bootstrap stability of this cluster (0.82) confirms it as a distinct and recurring clinical entity. A notable exception in our cohort was an ambulatory patient with Congenital Myasthenic Syndrome. With a negative silhouette width (−0.13), this individual represented a mathematical boundary case between neighbouring clusters. Preserved mobility and spontaneous breathing favoured assignment toward the stable neuromuscular phenotype (Cluster 1), whereas severe bulbar dysfunction, chronic *Pseudomonas aeruginosa* colonization, and irreversible structural lung damage resembled the dysphagia‐associated high‐risk phenotypes. Rather than weakening the clustering solution, this case highlights an important clinical insight: in selected conditions such as congenital myasthenic syndrome, gross motor function may not adequately reflect airway safety or pulmonary risk. By clustering patients solely on clinical parameters independent of radiological findings and subsequently mapping CT abnormalities onto these groups, we identified clinically meaningful phenotypes associated with distinct patterns of structural lung disease. These findings suggest that isolated variables such as ventilation mode are insufficient to capture pulmonary risk, whereas the interaction of bulbar dysfunction and impaired airway clearance may be more informative. Besides the described pathologies of the respiratory system additional respiratory pathologies following rib cage deformity and scoliosis were detected. In three patients, these skeletal deformities resulted in a compression of the bronchial or tracheal airway. This obstruction of the large airways may further impair regional ventilation [24, 25] and mucociliary clearance of airway secretions [26]. In addition, airway obstruction may cause chronic consolidation as a cause of a persistent infectious focus [27]. Thus, most subjects in this cohort exhibited significant pulmonary pathologies identified by chest CT scan, necessitating therapeutic intervention. Our data also suggest a potential diagnostic gap of conventional chest radiography in this high‐risk population. In a retrospective sub‐analysis, the CT abnormality associated with subsequent management change was not clearly identifiable on the preceding radiograph in 60% of evaluable cases. This observation should be considered hypothesis‐generating rather than definitive evidence of CT superiority. Within the constraints of the ALARA (As Low As Reasonably Achievable) principle, chest CT may therefore be considered in selected patients when clinically relevant findings remain insufficiently explained after standard assessment, including plain radiography. The objective identification of bronchiectasis and chronic consolidation in children with severe neurological disorders, in addition to the impaired mucociliary clearance, has a great impact on the management of respiratory disease [28, 29]. Importantly, published management guidelines for pediatric bronchiectasis recommend consistent, resistance‐guided antibiotic treatment of pulmonary exacerbations [5]. Pulmonary exacerbations may be prevented with the consequent application of airway clearance techniques and chest physiotherapy in the infection‐free status. Furthermore, if frequent pulmonary exacerbations are present, the diagnosis of non‐CF bronchiectasis gives a rationale to suppress colonizing bacteria with inhaled or systemic suppressive antibiotic therapies. Although awareness of respiratory morbidity in this population has increased and specific management recommendations have recently emerged, the radiological confirmation of structural lung disease in clinical practice still frequently serves as the decisive trigger for intensifying pulmonary therapies [30, 31]. However, we must caution against delaying the initiation of airway clearance until irreversible structural damage is proven by CT. As demonstrated by our clinical phenotypes, highly vulnerable patients, particularly those combining severe motor impairment with dysphagia (Cluster 2), require proactive preventive measures based on clinical risk factors long before bronchiectasis manifests [6, 32]. While our descriptive findings support the clinical rationale for early airway management, this study does not demonstrate that proactive interventions change long‐term structural outcomes. Prospective, controlled trials are required to prove the protective efficacy of early intervention. Several further limitations should be acknowledged. First, the retrospective single‐center design and tertiary care setting limit generalizability and likely enriched the cohort for children with complex disease trajectories and clinically suspected respiratory involvement. Second, patient selection was symptom‐driven, which predisposes to overrepresentation of structural abnormalities and precludes any inference on prevalence. Third, CT protocols varied over the long inclusion period. Although intravenous contrast was used in a minority of cases, this was unlikely to have materially influenced the identification of the major structural abnormalities assessed in this study. Temporal changes in imaging practice and respiratory management could also not be assessed systematically. Fourth, relevant clinical variables were derived from existing records and may have been incompletely documented. The mere prescription or availability of mechanical in‐exsufflation devices in our cohort does not inherently guarantee optimal utilization. The effective application of home respiratory therapies is highly complex and can be limited by numerous external barriers rather than a lack of caregiver commitment. Recent literature highlights that the real‐world usage of non‐invasive respiratory devices is significantly influenced by socioeconomic determinants, disparities in healthcare access, and substantial equipment‐related challenges, including physical discomfort and a lack of pediatric‐specific interfaces [33, 34]. Fifth, the clustering approach and the application of unsupervised machine learning in this small cohort were exploratory and require validation in larger independent populations. Finally, because chest CT was used cautiously in view of radiation exposure, imaging was generally reserved for selected clinical situations, which may have further contributed to selection bias. Despite these limitations, our findings support the hypothesis that dysphagia, impaired airway clearance, microbial colonization, and thoracic restriction interact in shaping structural lung disease in this vulnerable population. These findings should not be interpreted as prevalence estimates and require prospective validation before broader imaging strategies can be recommended.

## Author Contributions

**Daniel A. F. Bernard:** conceptualization, investigation, writing – original draft, methodology, visualization, writing – review and editing, software, formal analysis, project administration, data curation. **Cathrin Dahl:** conceptualization, investigation, writing – review and editing, methodology, formal analysis, data curation. **Luca Salhöfer:** data curation, investigation, writing – review and editing, methodology, formal analysis. **Anna Pauly:** writing – review and editing, Investigation, methodology. **Mathis Steindor:** methodology, writing – review and editing, investigation, conceptualization. **Margarete Olivier:** methodology, writing – review and editing, investigation. **Ulrike Schara‐Schmidt:** methodology, writing – review and editing, investigation. **Matthias Welsner:** methodology, writing – review and editing, conceptualization, investigation. **Bernd Schweiger:** methodology, writing – review and editing, resources, data curation, investigation. **Sebastian Zensen:** methodology, writing – review and editing, resources, investigation. **Marcel A. Drews:** methodology, writing – review and editing, investigation. **Johannes Haubold:** methodology, writing – review and editing, resources, investigation. **Michael Forsting:** methodology, writing – review and editing, resources, supervision. **Lale Umutlu:** methodology, writing – review and editing, resources, supervision. **Marcel Opitz:** conceptualization, investigation, writing – original draft, methodology, writing – review and editing, visualization, formal analysis, project administration, resources, supervision. **Florian Stehling:** methodology, writing – review and editing, resources, supervision, project administration, formal analysis, conceptualization, investigation, writing – original draft.

## Funding

The authors have nothing to report.

## Ethics Statement

This retrospective study was approved by the Ethics Committee of the Medical Faculty of the University of Duisburg‐Essen, Germany (reference number 25‐12617‐BO). All procedures performed in the study involving human participants were conducted in accordance with the ethical standards of the Ethics Committee of the Medical Faculty of the University of Duisburg‐Essen and with the 1964 Declaration of Helsinki and its later amendments. The requirement for informed consent was waived due to the retrospective and anonymised nature of the data analysis.

## Conflicts of Interest

The authors declare no conflicts of interest.

## Supporting information


**E‐Figure 1. Validation of cluster stability and individual patient assignment.** Silhouette plot of the identified phenotypes. The overall average silhouette width (ASW, dashed line) is 0.52. ASW values for Cluster 1, Cluster 2, and Cluster 3 are 0.61, 0.68, and 0.33, respectively. The most negative value identifies a patient with Congenital Myasthenic Syndrome showing a combination of preserved mobility and severe bulbar dysfunction.

**E‐Figure 2. The diagnostic gap of conventional chest radiographs (CXR).** Flowchart summarizing the timing and clinical relevance of CXR preceding CT in the study cohort (n = 34). In the subgroup of patients (n = 22) where CXR was performed within 30 days prior to CT, the CT exam prompted a change in clinical management in 91% of cases. A retrospective re‐review of these 20 cases revealed that in 60%, the triggering abnormality was not clearly identifiable on the preceding CXR.

**E‐Table 1:** Summarizes the spectrum of underlying diseases. Most patients had neuromuscular disorders, including congenital myopathies, motor neuron diseases, and muscular dystrophies. Other diagnoses included cerebral palsy, epileptic encephalopathies, congenital malformations and inflammatory CNS disorders.

## Data Availability

The data that support the findings of this study are available on request from the corresponding author. The data are not publicly available due to privacy or ethical restrictions. The data that support the findings of this study are available from the corresponding author upon reasonable request.

## References

[1] M. Toussaint , O. van Hove , D. Leduc , et al., “Invasive Versus Non‐Invasive Paediatric Home Mechanical Ventilation: Review of the International Evolution over the Past 24 Years,” Thorax 79, no. 6 (2024): 581–588. 38365452 10.1136/thorax-2023-220888

[2] S. Guerin , C. Bieli , R. Corbelli , et al., “Registry‐Based Surveillance of Paediatric Home Respiratory Support in Switzerland: Methodology and Initial Findings,” Swiss Medical Weekly 155, no. 6 (2025): 4193. 40570248 10.57187/s.4193

[3] D. P. Escobar‐Serna , J. S. Barajas‐Romero , J. J. Peralta‐Palmezano , et al., “OP029 Topic: AS15–Lung: Respiratory Support/Acute Respiratory Failure/Other: Pediatric Non‐Invasive Respiratory Support Failure: Risk Factors & Outcomes in Latin America,” Pediatric Critical Care Medicine: A Journal of The Society of Critical Care Medicine and the World Federation of Pediatric Intensive and Critical Care Societies 25, no. 11S (2024): e11–e11.

[4] J. Florén , M. Ekström , B. Lindahl , A. Markström , A. Palm , and Å. Israelsson‐Skogsberg , “Swedish National Cohort of Children Living With Long‐Term Respiratory Support (DISCOVERY‐P): Cohort Profile,” BMJ Open 15, no. 4 (2025): e090241. 10.1136/bmjopen-2024-090241 PMC11997812 40228848

[5] P. C. Seddon , “Respiratory Problems in Children With Neurological Impairment,” Archives of Disease in Childhood 88, no. 1 (2003): 75–78. 12495971 10.1136/adc.88.1.75 PMC1719284

[6] J. Hull , R. Aniapravan , E. Chan , et al., “British Thoracic Society Guideline for Respiratory Management of Children With Neuromuscular Weakness,” Thorax 67, no. Suppl 1 (2012): i1–i40. 22730428 10.1136/thoraxjnl-2012-201964

[7] H. B. Panitch , “Respiratory Implications of Pediatric Neuromuscular Disease,” Respiratory Care 62, no. 6 (2017): 826–848. 28546380 10.4187/respcare.05250

[8] M. Chatwin , M. Toussaint , M. R. Gonçalves , et al., “Airway Clearance Techniques in Neuromuscular Disorders: A State of the Art Review,” Respiratory Medicine 136 (2018): 98–110. 29501255 10.1016/j.rmed.2018.01.012

[9] C. E. Erasmus , K. van Hulst , J. J. Rotteveel , M. A. A. P. Willemsen , and P. H. Jongerius , “Clinical Practice: Swallowing Problems in Cerebral Palsy,” European Journal of Pediatrics 171, no. 3 (2012): 409–414. 21932013 10.1007/s00431-011-1570-y PMC3284655

[10] L. van den Engel‐Hoek , C. E. Erasmus , K. C. M. van Hulst , J. C. Arvedson , I. J. M. de Groot , and B. J. M. de Swart , “Children With Central and Peripheral Neurologic Disorders Have Distinguishable Patterns of Dysphagia on Videofluoroscopic Swallow Study,” Journal of Child Neurology 29, no. 5 (2014): 646–653. 24022110 10.1177/0883073813501871

[11] A. B. Chang , A. Bush , and K. Grimwood , “Bronchiectasis in Children: Diagnosis and Treatment,” Lancet 392, no. 10150 (2018): 866–879. 30215382 10.1016/S0140-6736(18)31554-X

[12] J. Grosse‐Onnebrink , J. Rudloff , C. Kessler , et al., “Acinetobacter Baumannii Is a Risk Factor for Lower Respiratory Tract Infections in Children and Adolescents With a Tracheostomy,” Pediatric Infectious Disease Journal 38, no. 10 (2019): 1005–1009. 31568139 10.1097/INF.0000000000002421

[13] J. Powell , S. Powell , M. W. Mather , et al., “Tracheostomy in Children Is Associated With Neutrophilic Airway Inflammation,” Thorax 78, no. 10 (2023): 1019–1027. 36808087 10.1136/thorax-2022-219557 PMC10511973

[14] F. Stehling , N. Pieper , A. Bouikidis , J. Steinmann , P.‐M. Rath , and U. Mellies , “Upper Airway Microbial Colonization in Patients With Neuromuscular Disorders,” Respirology 21, no. 7 (2016): 1285–1291. 27221716 10.1111/resp.12814

[15] A. B. Chang , R. Fortescue , K. Grimwood , et al., “European Respiratory Society Guidelines for the Management of Children and Adolescents With Bronchiectasis,” European Respiratory Journal 58, no. 2 (2021): 2002990. 33542057 10.1183/13993003.02990-2020

[16] A. B. Chang , J. W. Upham , I. B. Masters , et al., “Protracted Bacterial Bronchitis: The Last Decade and the Road Ahead,” Pediatric Pulmonology 51, no. 3 (2016): 225–242. 26636654 10.1002/ppul.23351 PMC7167774

[17] L. Boel , K. Pernet , M. Toussaint , et al., “Respiratory Morbidity in Children With Cerebral Palsy: An Overview,” Developmental Medicine and Child Neurology 61, no. 6 (2019): 646–653. 30320434 10.1111/dmcn.14060

[18] C. A. Gerdung , A. Tsang , A. S. Yasseen , K. Armstrong , H. J. McMillan , and T. Kovesi , “Association Between Chronic Aspiration and Chronic Airway Infection With Pseudomonas Aeruginosa and Other Gram‐Negative Bacteria in Children With Cerebral Palsy,” Lung 194, no. 2 (2016): 307–314. 26883134 10.1007/s00408-016-9856-5

[19] F. Stehling , C. Dohna‐Schwake , U. Mellies , and J. Große‐Onnebrink , “Decline in Lung Volume With Duchenne Muscular Dystrophy Is Associated With Ventilation Inhomogeneity,” Respiratory Care 60, no. 9 (2015): 1257–1263. 25944942 10.4187/respcare.04025

[20] M. Steindor , A. Pichler , L. Heitschmidt , et al., “Multiple Breath Washout Lung Function Reveals Ventilation Inhomogeneity Unresponsive to Mechanical Assisted Cough in Patients With Neuromuscular Disease,” BMC Pulmonary Medicine 22, no. 1 (2022): 217. 35659287 10.1186/s12890-022-02012-z PMC9166427

[21] M. Estenne , P. A. Gevenois , W. Kinnear , P. Soudon , A. Heilporn , and A. De Troyer , “Lung Volume Restriction in Patients With Chronic Respiratory Muscle Weakness: The Role of Microatelectasis,” Thorax 48, no. 7 (1993): 698–701. 8153916 10.1136/thx.48.7.698 PMC464647

[22] A. b Chang , G. j Redding , and M. l Everard , “Chronic Wet Cough: Protracted Bronchitis, Chronic Suppurative Lung Disease and Bronchiectasis,” Pediatric Pulmonology 43, no. 6 (2008): 519–531. 18435475 10.1002/ppul.20821

[23] B. Hov , T. Andersen , M. Toussaint , et al., “Prevalence of Long‐Term Mechanical Insufflation‐Exsufflation in Children With Neurological Conditions: A Population‐Based Study,” Developmental Medicine and Child Neurology 63, no. 5 (2021): 537–544. 33393110 10.1111/dmcn.14797 PMC8048789

[24] T. M. Andrews , C. M. Myer , and S. P. Gray , “Abnormalities of the Bony Thorax Causing Tracheobronchial Compression,” International Journal of Pediatric Otorhinolaryngology 19, no. 2 (1990): 139–144. 2197245 10.1016/0165-5876(90)90219-h

[25] L. F. Donnelly and G. S. Bisset , “Airway Compression in Children With Abnormal Thoracic Configuration,” Radiology 206, no. 2 (1998): 323–326. 9457181 10.1148/radiology.206.2.9457181

[26] M. Qiabi , K. Chagnon , A. Beaupré , J. Hercun , and G. Rakovich , “Scoliosis and Bronchial Obstruction,” Canadian Respiratory Journal 22, no. 4 (2015): 206–208. 26083538 10.1155/2015/640573 PMC4530852

[27] S. Schena and A. S. Krupnick , “Recurrent Empyema Secondary to Persistent Spinal Compression of the Left Main Bronchus,” Journal of Thoracic and Cardiovascular Surgery 145, no. 1 (2013): 294. 22939853 10.1016/j.jtcvs.2012.08.005

[28] A. B. Chang , S. C. Bell , P. J. Torzillo , et al., “Chronic Suppurative Lung Disease and Bronchiectasis in Children and Adults in Australia and New Zealand Thoracic Society of Australia and New Zealand Guidelines,” Medical Journal of Australia 202, no. 1 (2015): 21–23. 25588439 10.5694/mja14.00287

[29] A. T Hill , A. L Sullivan , J. D Chalmers , et al., “British Thoracic Society Guideline for Bronchiectasis in Adults,” Thorax 74, no. Suppl 1 (2019): 1–69. 10.1136/thoraxjnl-2018-212463 30545985

[30] J. Legg , J.‐L. Allen , M. Andrew , et al., “BTS Clinical Statement on the Prevention and Management of Community‐Acquired Pneumonia in People With Learning Disability,” Thorax 78, no. Suppl 1 (2023): s1.1‐s31. 10.1136/thorax-2022-219698 36863773

[31] M. D. Mauritz , U. von Both , C. Dohna‐Schwake , et al., “Clinical Recommendations for the Inpatient Management of Lower Respiratory Tract Infections in Children and Adolescents With Severe Neurological Impairment in Germany,” European Journal of Pediatrics 183, no. 3 (2024): 987–999. 38172444 10.1007/s00431-023-05401-6 PMC10951000

[32] D. J. Birnkrant , K. Bushby , C. M. Bann , et al., “Diagnosis and Management of Duchenne Muscular Dystrophy, Part 2: Respiratory, Cardiac, Bone Health, and Orthopaedic Management,” Lancet Neurology 17, no. 4 (2018): 347–361. 29395990 10.1016/S1474-4422(18)30025-5 PMC5889091

[33] M. Hurvitz , K. Sunkonkit , A. Defante , et al., “Non‐Invasive Ventilation Usage and Adherence in Children and Adults With Duchenne Muscular Dystrophy: A Multicenter Analysis,” Muscle and Nerve 68, no. 1 (2023): 48–56. 37226876 10.1002/mus.27848

[34] D. Olmstead , A. Carroll , J. Klein , and J. E. MacLean , “The Experience of Children Using Long‐Term Non‐Invasive Ventilation: A Qualitative Study,” Frontiers in Sleep 3 (2024): 1459349. 41424490 10.3389/frsle.2024.1459349 PMC12713816

