# Supplementary figures and images for: Structural Lung Disease in Children and Adolescents With Severe Neurological Disorders
Source: Pediatr Pulmonol. 2026 Jun 10;61(6):e71698. doi: 10.1002/ppul.71698 (PMC13254491; doi:10.1002/ppul.71698)

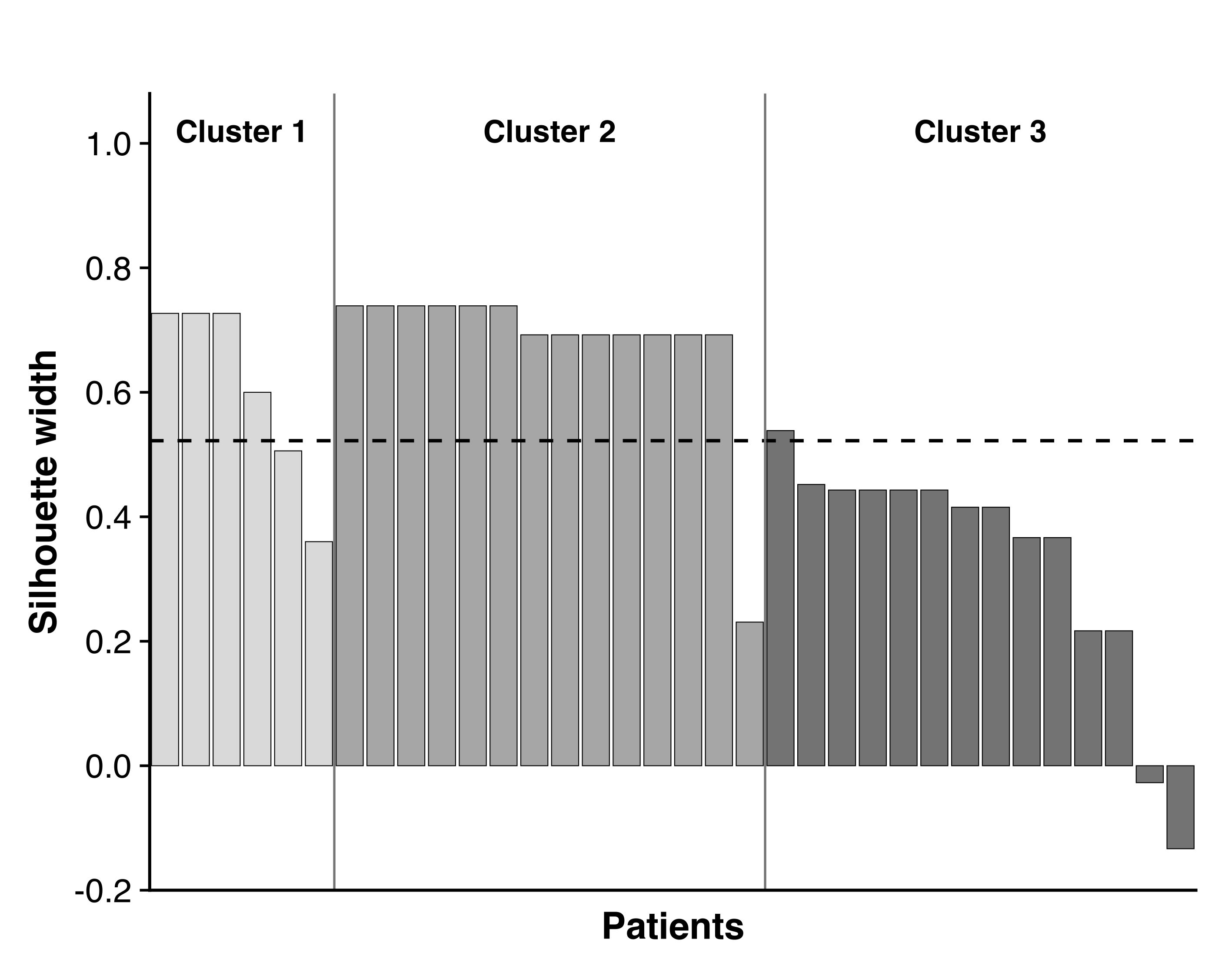

Supplement: Supplementary file 1 — E‐Figure 1. Validation of cluster stability and individual patient assignment. Silhouette plot of the identified phenotypes. The overall average silhouette width (ASW, dashed line) is 0.52. ASW values for Cluster 1, Cluster 2, and Cluster 3 are 0.61, 0.68, and 0.33, respectively. The most negative value identifies a patient with Congenital Myasthenic Syndrome showing a combination of preserved mobility and severe bulbar dysfunction. [file PPUL-61-0-s002.tiff]

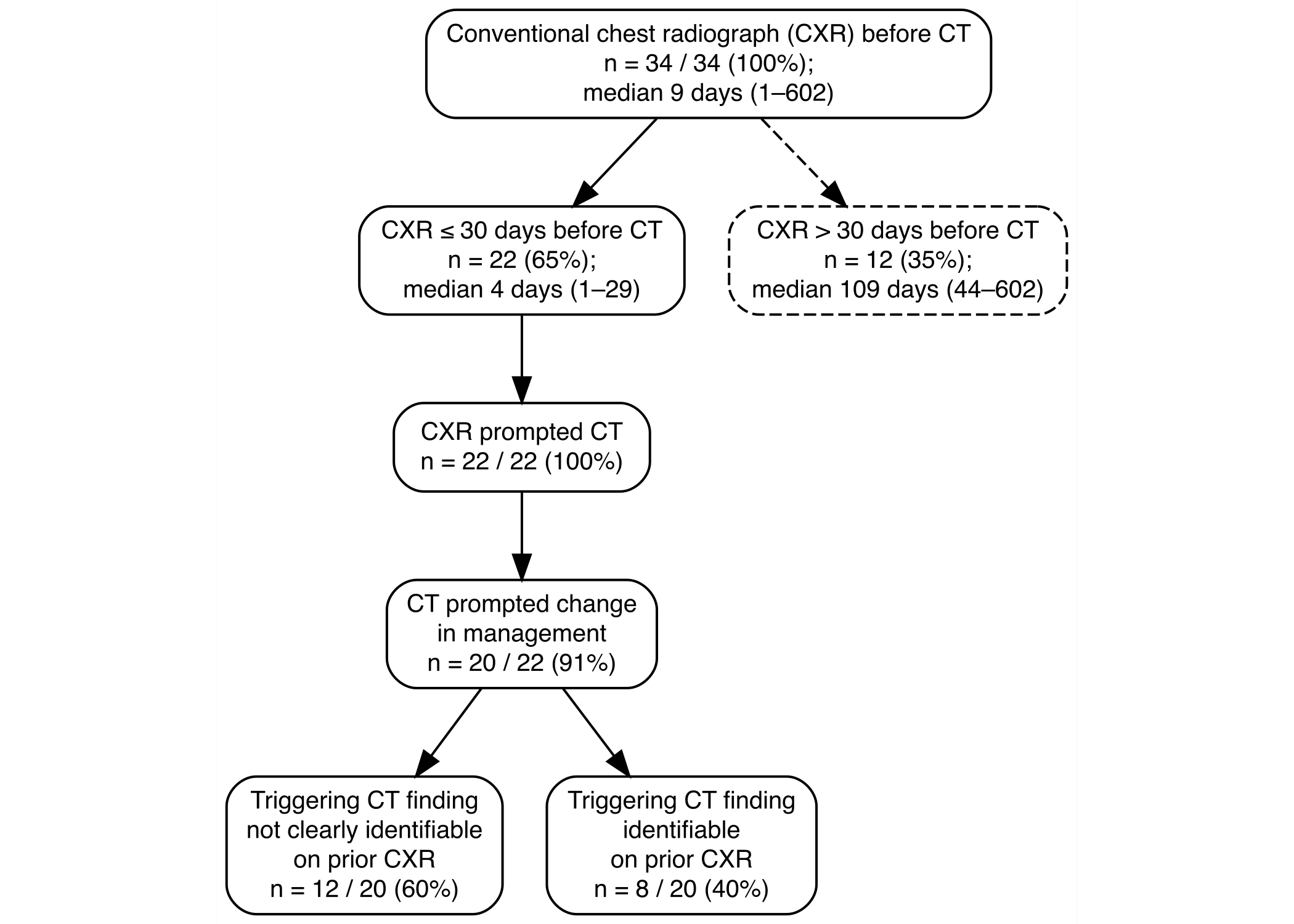

Supplement: Supplementary file 2 — E‐Figure 2. The diagnostic gap of conventional chest radiographs (CXR). Flowchart summarizing the timing and clinical relevance of CXR preceding CT in the study cohort (n = 34). In the subgroup of patients (n = 22) where CXR was performed within 30 days prior to CT, the CT exam prompted a change in clinical management in 91% of cases. A retrospective re‐review of these 20 cases revealed that in 60%, the triggering abnormality was not clearly identifiable on the preceding CXR. [file PPUL-61-0-s003.tif]
