# Supplementary material for: Structural Lung Disease in Children and Adolescents With Severe Neurological Disorders
Source: Pediatr Pulmonol. 2026 Jun 10;61(6):e71698. doi: 10.1002/ppul.71698 (PMC13254491; doi:10.1002/ppul.71698)
Supplement: Supplementary file 3 — E‐Table 1: Summarizes the spectrum of underlying diseases. Most patients had neuromuscular disorders, including congenital myopathies, motor neuron diseases, and muscular dystrophies. Other diagnoses included cerebral palsy, epileptic encephalopathies, congenital malformations and inflammatory CNS disorders. [file PPUL-61-0-s001.docx]

**E-table 1** summarizes the spectrum of underlying diseases. Most patients had neuromuscular disorders, including congenital myopathies, motor neuron diseases, and muscular dystrophies. Other diagnoses included cerebral palsy, epileptic encephalopathies, congenital malformations and inflammatory CNS disorders.

|  | **E-table 1**:  Spectrum of underlying diseases |  |
| --- | --- | --- |
|  | **n** | **detailed diagnosis** |
| **neuromuscular disorders** |  |  |
| Muscular dystrophies | 2 | duchenne muscular dystrophy, congenital myotonic dystrophy type I |
| Congenital myopathies | 11 | infantile Pompe disease, pompe disease (2), BAG3 associated myofibrillar myopathy, MTM1-myotubular myopathy (3), nemaline myopathy (2), mitochondriopathy, congenital myasthenia (CHRMF) |
| Motor neuron diseases | 7 | juvenile ALS, SMA type I, SMA type II (3), SMA type IIIA, infantile spinal atrophy of muscles with weakness of diaphragm (SMARD1) |
| Unclear neuromuscular | 1 | unknown neuromuscular disease |
| **other neurological disorders** |  |  |
| Cerebral palsy with epilepsia | 2 | cerebral palsy with epilepsy, cerebral palsy with Lennox-Gastaut syndrome |
| Epileptic encephalopathies | 2 | epileptic encephalopathy (STXBP1), epileptic encephalopathy (NTRK2) |
| Congenital malformation syndromes | 7 | unknown syndromal disease (4), complex malformations of brain, chromosomal anomaly with complex malformations of brain, CHARGE syndrome |
| Inflammatory CNS disorders | 2 | post-meningitic hydrocephalus, transverse myelitis |
| Total | 34 | — |
